# Lags in the provision of obstetric services to indigenous women and their implications for universal access to health care in Mexico

**DOI:** 10.1080/26410397.2020.1778153

**Published:** 2020-08-06

**Authors:** Clara Juárez-Ramírez, Gustavo Nigenda, Alma L. Sauceda-Valenzuela, Aremis Villalobos

**Affiliations:** a Researcher, Centre for Health Systems Research, National Institute of Public Health, México City, México; b Professor, National School of Nursing and Obstetrics, National Autonomous University of Mexico, Mexico City, Mexico. Correspondence: gnigenda@outlook.com

**Keywords:** universal health coverage, indigenous women, access, obstetric services, social determinants of health, Mexico

## Abstract

Through quantitative and qualitative methods, in this article the authors describe the perspectives of indigenous women who received antenatal and childbirth medical care within a care model that incorporates a non-governmental organisation (NGO), Partners in Health. They discuss whether the NGO model better resolves the care-seeking process, including access to health care, compared with a standard model of care in government-subsidised health care units (setting of health services networks). Universal health coverage advocates access for the most disadvantaged and vulnerable populations as a priority. However, the issue of access includes problems related to the effect of certain structural social determinants that limit different aspects of the obstetric care process. The findings of this study show the need to modify the structure of organisational values in order to place users at the centre of medical care and ensure respect for their rights. The participation of agents outside the public system, such as NGOs, can be of great value for moving in this direction. Women’s participation is also necessary for learning how they are being cared for and the extent to which they are satisfied with obstetric services. This research experience can be used for other countries with similar conditions.

## Résumé

Au moyen de méthodes quantitatives et qualitatives, les auteurs décrivent dans cet article les perspectives des femmes autochtones qui ont reçu des soins médicaux prénatals et obstétricaux dans le cadre d’un modèle de soins qui associe une organisation non gouvernementale (l’ONG Partners in Health). Ils se demandent si le modèle de l’ONG est mieux à même de résoudre le processus de demande de soins, notamment l’accès aux soins de santé, par rapport au modèle type de soins dans les services de santé financés par l’État (environnement de réseaux de services de santé). La couverture santé universelle permet de donner accès en priorité aux populations les plus défavorisées et vulnérables. Néanmoins, la question de l’accès comporte des problèmes relatifs à l’effet de certains déterminants sociaux structurels qui limitent différents aspects du processus de soins obstétricaux. Les conclusions de cette étude montrent la nécessité de modifier la structure des valeurs organisationnelles afin de placer les usagers au centre des soins médicaux et garantir le respect de leurs droits. La participation des agents extérieurs au système public, comme les ONG, peut être précieuse pour aller dans cette direction. La participation des femmes est aussi nécessaire pour apprendre quel traitement elles reçoivent et dans quelle mesure elles sont satisfaites des services obstétricaux. L’expérience de cette recherche peut être utilisée pour d’autres pays placés dans des conditions similaires.

## Resumen

Por medio de métodos cuantitativos y cualitativos, los autores de este artículo describen las perspectivas de las mujeres indígenas que recibieron atención prenatal y atención médica del parto dentro de un modelo de atención que incorpora una organización no gubernamental (la ONG *Partners in Health*). Analizan si el modelo de la ONG resuelve mejor el proceso de búsqueda de atención, que incluye el acceso a los servicios de salud, comparado con el modelo de atención estándar en las unidades de salud subsidiadas por el gobierno (ámbito de las redes de servicios de salud). La cobertura médica universal aboga por el acceso para las poblaciones más desatendidas y vulnerables como una prioridad. Sin embargo, el asunto de acceso implica problemas relacionados con el efecto de ciertos determinantes estructurales sociales que limitan diferentes aspectos del proceso de obtención de cuidados obstétricos. Los hallazgos de este estudio muestran la necesidad de modificar la estructura de valores institucionales a fin de colocar a las usuarias al centro de la atención médica y garantizar respeto por sus derechos. La participación de agentes fuera del sistema público, como las ONG, puede ser de gran valor para avanzar en esta dirección. Además, la participación de las mujeres es necesaria para saber cómo están siendo atendidas y en qué medida están satisfechas con los servicios obstétricos. Esta experiencia de investigación puede utilizarse en otros países con condiciones similares.

## Introduction

In the Latin America (LATAM) region, characterised as one of the most unequal regions in the world,^1–3^ health care systems are facing enormous challenges in meeting the health needs of the most disadvantaged populations.^3^ These populations include indigenous groups, who continue to have insufficient access to health services, thus representing a social inequity as yet unresolved.^4^ Recent assessments conducted by the World Health Organization (WHO) regarding the global response towards commitments to improving the health of the population have recognised that the outstanding challenge for the present century remains universal access to health care services.^5,6^ Universal access is not a health policy that can only be implemented in wealthy countries;^7^ it is also a programmatic and ideological approach that goes hand in hand with the countries’ economic development models.^8^ However, the shortfall in universal access to health occurs mainly in poor countries, where the most vulnerable populations continue to have the worst health indicators.^3,9,10^

In LATAM, health system reforms have been implemented for various purposes, including the expansion of health care coverage and the reduction of out-of-pocket expenses for health care.^11^ Multiple strategies have also been developed to improve the provision of health care services, one of them being the creation of integrated health care networks, proposed by the Pan American Health Organization (PAHO).^12^ These networks seek to strengthen the response of health care systems, whose infrastructure and human resource capacity is often insufficient.^13^ Non-governmental organisations (NGOs) also participate in the provision of services to the most impoverished populations. Some countries recognise them as an important part of the health system,^14^ while others do not include NGOs in their governance model,^15^ and yet, in practice, they are part of the social support network of vulnerable populations.

In Mexico, the government has implemented a two-fold strategy to address the reproductive health needs of the population. On the one hand, it developed a universal coverage strategy based on the expansion of public insurance, which provided funding to both health centres and hospitals to encourage the responsiveness of medical services in the health care system.^16^ In addition, the Ministry of Health promoted a patient-centred care model, fostering a respectful regard for the reproductive rights of users. This model was supported by the National Centre for Gender Equity and Reproductive Health, a federal agency responsible for promoting good practices in pregnancy, childbirth and postnatal care. Yet, although it is now possible to identify cases of full implementation,^17^ progress is slow.^18^

The case of sexual and reproductive health care for indigenous women is a powerful exemplar. Despite institutional efforts to reduce complications during pregnancy, a decrease in maternal deaths has not yet been achieved. In 2018, there were 667 maternal deaths in Mexico,^19^ the majority (77%) in Chiapas, a state with a high percentage of indigenous people. The available evidence suggests that various environmental and cultural circumstances interact around this problem, in addition to insufficient access to the health care system and to the use of medical services.^20^ Services show significant technical deficiencies in terms of care*,* generating risks for indigenous women due to the uncertain resolution of problems during antenatal and delivery care.^21^

To monitor progress in this respect, we documented two models of obstetric care, in order to understand: what hurdles in medical care hinder access and lead to complications during pregnancy and childbirth that result in maternal deaths, and what universal access to health care services means in the indigenous context. We believe the answers to these questions will provide very relevant evidence for reorientating the organisation of health care services for indigenous women.

## Methods

### Study design

We tested the hypothesis that indigenous women who received antenatal and delivery care in a model that incorporated an NGO (*Partners in Health:* www.pih.org) were more able to navigate the process of seeking care, including access, compared to a standard model of care prevalent in government-subsidised health care units and in the setting of health services networks. With this in mind, we explored the daily difficulties of both service-users (indigenous women) and service-providers, in order to identify the hurdles or obstacles to the smooth passage of women through the different levels of medical care (continuity of care).

The study design was cross-sectional and observational. Two models of medical care offered to rural and indigenous communities were selected: one, in the state of Chiapas, included the participation of an NGO; while the other, in the state of Oaxaca, followed a standard model of care.

We used quantitative and qualitative methods for data collection,^22,23^ with targeted sampling of (a) women of indigenous origin and (b) health care personnel (nurses, doctors, social workers, health workers, among others). The quantitative methods sought to make a socio-demographic characterisation of the study population and obtain primary data, since these regions lack specific relevant information on the research topic. The qualitative component elaborated on women’s difficulties in accessing emergency obstetric care. In the case of health care personnel, we documented the problematic knots or obstacles they identified to providing quality medical care to indigenous women, as well as their degree of job satisfaction.

The inclusion criteria for *indigenous women* were: being over 18 years old; speaking an indigenous language or having indigenous parents or grandparents (ethnicity criteria); and having had at least one delivery/birth in the last five years. For *health care personnel,* a professional from each area involved in some part of the obstetric care process was selected.

The two study sites were: the Mixteca region of the state of Oaxaca for the public/government service model; and the Frailesca region, in the state of Chiapas, for the service model with NGO participation.

### Data collection

Information was collected in two stages during 2017–2018: in four indigenous communities in Oaxaca, served by five primary health care units and two secondary level hospitals; and in five communities in Chiapas, served by five primary health care units, one secondary level hospital, and one maternal health clinic.

For the quantitative stage, following review of the academic literature on the study problem, two types of questionnaire were developed, one for health care personnel (self-administered), with six topics (86 questions); and one for indigenous women, with seven topics (176 questions). In the qualitative stage, the interview guide for women explored five topics: family composition; obstetric history; dignified treatment; care for pregnancy and childbirth; and quality of care. The interview guide for health care personnel explored two topics: the maternal health care process (training, medical care, postpartum and newborn care); and quality of care (access, user treatment, infrastructure, antenatal and postnatal examination). All the interviews were audio recorded.

In both stages, participants were recruited in the medical (community and hospital) units, by direct invitation. Selection of women for the qualitative stage was made via screening questions incorporated into the questionnaire. In the case of health care personnel, all those who agreed to participate were interviewed (Table 1).

**Table 1.** Collected data

Type interviewed	Chiapas	Oaxaca	Total
Health care personnel	Questionnaires: 30	Questionnaires: 31	61
	Qualitative interviews: 25	Qualitative interviews: 24	49
Indigenous women	Questionnaires: 145	Questionnaires: 149	294
	Qualitative interviews: 28	Qualitative interviews: 27	55
		Total questionnaires both states	**355**
		Total qualitative interviews both states	**104**

Source: Personal research based on the results.

### Data analysis

For quantitative data, Stata 12.0 SE software was used. A descriptive analysis was carried out; the results were stratified by model of medical care (Oaxaca vs Chiapas) presenting absolute and relative frequencies. For health care personnel the following subjects were analysed: sociodemographic characteristics of health care providers and of the medical unit; type of limitations for the provision of antenatal consultations; and type of communication used with women who only speak an indigenous language. For female health care users, the following were analysed: sociodemographic characteristics; antenatal and postnatal care during last pregnancy; and perceptions about care quality. Chi2 tests were performed to assess independence between the variables of interest of the Chiapas and Oaxaca models. In the case of health care personnel, those variables were: level of training for activities related to the provision of antenatal consultation; infrastructure availability; human resources; organisational aspects of high-risk pregnancy care and obstetric emergencies; referral of pregnant patients and women with obstetric emergencies; knowledge regarding the availability of supplies and of study required in antenatal and childbirth care, according to official regulations. In the case of female health care users, the variable of interest was care quality, which was analysed through the difference statistic of ratios between states on knowledge of the actions required in antenatal care, also in accordance with the regulations.

Analysis of qualitative data was performed manually. Interviews were transcribed and coded according to the microanalysis technique (Grounded Theory); ^24,25^ open coding was carried out based on topics included in the interview guide, and information summaries were prepared. This article only presents the results for Topic IV, care during pregnancy and childbirth, from the interviews with women. From the interviews with health care personnel, we only present the results for Topic II, maternal health care process.*

### Ethical aspects

Standards from the Helsinki Declaration^27^ were taken into consideration. Oral and written informed consent was obtained. The study protocol was approved by the Ethics and Research Commissions of the National Institute of Public Health of Mexico: CI1416-2016, on 6 September 2016. The names of the medical institutions participating in the study have been omitted to preserve the anonymity of the participants.

## Results

### Profile of the respondents

*Users*: 294 users of health care services were surveyed, 145 in Chiapas and 149 in Oaxaca. More than half of the users were between 20–29 years old and about a third of them were between 30–39 years old. Most of them lived in a stable relationship, as a couple, and were homemakers (Table 2).

**Table 2.** Sociodemographic characteristics of the women interviewed

	Chiapas (*n *= 145)		Oaxaca (*n *= 149)		Total (*n *= 294)	
	*n*	%	*n*	%	*n*	%
**Age group**						
15–19	21	14.5	16	10.7	37	12.6
20–29	83	57.2	80	53.7	163	55.4
30–39	35	24.1	48	32.2	83	28.2
40-45	6	4.1	5	3.4	11	3.7
**Marital status**						
Single	4	2.8	18	12.1	22	7.5
Married/cohabiting	136	93.8	125	83.8	261	88.8
Separated/divorced	4	2.8	5	3.4	9	3.1
Widowed	1	0.7	1	0.7	2	0.7
**Literacy**						
Yes	134	92.4	148	99.3	282	95.9
No	11	7.6	1	0.7	12	4.1
**Occupation**						
Home/housewife	141	97.9	124	83.2	265	90.4
Household employee	0	0	5	3.4	5	1.7
Merchant/salesperson	0	0	13	8.7	13	4.4
Employee public/private sector	2	1.4	4	2.7	6	2
Works in the fields	1	0.7	1	0.7	2	0.7
Student	0	0	1	0.7	1	0.3
Other	0	0	1	0.7	1	0.3
**Identifies herself as an indigenous person**						
Yes	2	1.4	112	75.2	114	38.8
No	143	98.6	37	24.8	180	61.2
**Speaks an indigenous language**						
Yes	1	0.7	62	41.6	63	21.4
No	144	99.3	87	58.4	231	78.6
**Any of her parents or grandparents speak an indigenous language**						
Yes	13	9	105	70.5	118	40.1
No	132	91	44	29.5	176	59.9
Indigenous Origin*						
Yes	13	9	142	95.3	155	52.7
No	132	91	7	4.7	139	47.3

* Including self-identification as an indigenous person or the interviewee or her parents or grandparents speak an indigenous language.

Source: Personal research based on the results.

*Health care personnel*: Sixty per cent of health providers in Chiapas and 52% of health providers in Oaxaca were women. Chiapas health providers were, on average, younger than those in Oaxaca (28.8 years versus 34 years). The majority of the personnel in Chiapas were nurses from the obstetrics and gynaecology department, medical interns, and general practitioners, while in Oaxaca the majority of the participants were personnel responsible for emergencies, followed by obstetrics medical staff, obstetrics nurses, medical assistants, and medical interns, among other positions. Almost 20% of the personnel in Oaxaca, and 17.8% in Chiapas, also served as director of the medical unit. Ninety-seven percent of the medical units in Chiapas belonged to state public health services, while in Oaxaca 70% belonged to public services, and 30% to another institution providing medical services for the federal social support program. Additionally, 67% of the units in Chiapas and 77% of the units in Oaxaca provided primary care; the rest in both states were secondary level, hospital care institutions (Table 3).

**Table 3.** Sociodemographic and occupational characteristics of the health care personnel in both models

	Chiapas (*n *= 30)		Oaxaca (*n *= 31)		Total (*n*=61)	
	*n*	%	*n*	%	*n*	%
**Sex**						
Female	17	60.7	14	51.9	31	56.4
Male	11	39.3	13	48.1	24	43.6
**Age**						
Average	27	28.8 years	24	34.0 years	51 years	31.2 years
Minimum		21 years		18 years		18 years
Maximum		46 years		58 years		58 years
**Position**						
Ward or on call obstetrician	0	0	2	7.1	2	3.7
Obstetrics and gynecology resident	0	0	1	3.6	1	1.9
Obstetrics and gynecology nurse	8	30.8	2	7.1	10	18.5
Medical assistant	3	11.5	2	7.1	5	9.3
Medical intern	6	23.1	2	7.1	8	14.8
Chief of Emergency	0	0	3	10.7	3	5.6
General practitioner	7	26.9	1	3.6	8	14.8
Other	2	7.7	15	53.6	17	31.4
**Director of the medical unit**						
Yes	5	17.9	6	19.4	11	18.6
No	23	82.1	25	80.6	48	81.4
**Institution of Affiliation**						
Institution providing medical services for the federal social support program	1	3.3	9	30.00	10	16.7
State public health services	29	96.7	21	70.00	50	83.3
**Level of care**						
Primary level	19	67.9	24	77.4	43	72.9
Secondary level	9	32.1	7	22.6	16	27.1

Source: Personal research based on the results.

### Obstetric care models and their efficiency

#### Standard model: government care

*Primary level care* is provided through community health centres. The number and type of health professionals providing medical consultation varies based on the size of the population in each town; consequently, some medical centres may even have departments of nutrition and dentistry, while smaller ones only have one office with a doctor and a nurse. There are also “health posts” (medicine dispensary run by community health workers); and mobile medical units (fully equipped trucks). (Figure S1 in Supplementary material describes the structure of the standard model.) At the community level, health workers, traditional midwives, and special project brigades identify newly pregnant women in order to start antenatal consultation during the first trimester. Of the women interviewed, 15% did not initiate antenatal care in the first trimester of pregnancy, and 28% had complications during pregnancy (Table 4).

**Table 4.** Characteristics of prenatal and postnatal care (last obstetric event)

	Chiapas (*n *= 145)		Oaxaca (*n *= 149)		Total (*n *= 294)	
	*n*	%	*n*	%	*n*	%
***Prenatal care***						
Had prenatal control medical consultations	142	97.9	130	95.6	272	96.8
Pregnancy trimester when she initiated prenatal visits						
1st trimester	122	87.1	109	84.5	231	85.9
2nd trimester	16	11.4	19	14.7	35	13
3rd trimester	2	1.4	1	0.8	3	1.1
Complications in last pregnancy	53	36.6	37	27.6	90	32.3
First pregnancy at the time of survey (primipara)	0	0	13	8.7	13	4.4
***Childbirth care*^a^**						
Point of care *						
Home (own or midwife’s)	29	20.0	3	2.2	32	11.3
State public health services	105	72.4	80	58.0	185	65.4
Institution providing medical services for the federal social support program	7	4.8	51	37.0	58	20.5
Social Security Institution	0	0.0	1	0.7	1	0.4
Private	3	2.1	3	2.2	6	2.1
Other	1	0.7	0	0.0	1	0.4
Birth resolution*						
Normal/vaginal	130	89.7	75	54.3	205	72.4
Scheduled caesarean	8	5.5	13	9.4	21	7.4
Emergency caesarean	7	4.8	47	34.1	54	19.1
Accompaniment in the birth process^b,*^	38	33.0	22	16.5	60	24.2
She was given information concerning the birth process^b^	53	45.7	60	45.5	113	45.6
Skin to skin contact at birth	85	58.6	72	52.9	157	55.9
***Post-obstetric event counselling***						
Postnatal depression*	34	24.0	17	13.0	51	18.0
Signs and symptoms of bleeding *	58	40.0	97	71.0	155	55.0
Preeclampsia-eclampsia*	82	71.0	114	86.0	196	70.0
Signs and symptoms of potential infections*	42	29.0	86	63.0	128	46.0
Thrombosis*	66	46.0	96	71.0	162	58.0
Nutrition*	80	56.0	93	68.0	173	62.0
Hand-washing*	89	62.0	107	79.0	196	70.0

^a^Excluding 13 cases of women whose first pregnancy was at the time of the survey

^b^Does not include home birth information

*P < 0.5 value, chi 2 independence test

Note: The difference in the percentage of C-sections in Chiapas versus Oaxaca may be attributable to the model of care followed by the NGO that favours the resolution of “natural birth” in the region studied in Chiapas in contrast to the standard care model subsidised by the government (Oaxaca), where the indication of caesarean section for the resolution of childbirth is usually more common. Likewise, this percentage of caesarean section for the Frailesca region of Chiapas does not necessarily reflect the general situation of the State, although it can be said that the State of Chiapas has presented a comparatively lower percentage of caesarean sections than Oaxaca in recent years (Chiapas 34% versus Oaxaca 45% in 2016).

Source: Personal research based on the results

Opinions of health care personnel surveyed in this regard were as follows: 26% indicated that women do not initiate timely antenatal care due to economic, language (13.3%), and cultural (13.3%) limitations, and to not habitually making decisions on their own (13.3%). When asked about activities they carry out to encourage women to attend antenatal visits at the beginning of pregnancy, 32% responded that they do not carry them out due to time constraints caused by excessive workload (32%), and lack of human resources in the medical unit (8%); lack of material resources (12%); difficulties in having private spaces to provide medical care (12%); and cultural differences or different values in the community regarding medical care (8%) (Table 5). In contrast, and compared to the opinions of the health personnel, the two main reasons women gave in the qualitative interviews for not going to the health centre were that it is often closed, and lengthy waiting times.

**Table 5.** Availability of infrastructure and personnel, and management and organisational aspects required in the care of high-risk pregnancies and obstetric emergencies, according to the opinion of health personnel in both Chiapas and Oaxaca models

	Chiapas (*n *= 30)		Oaxaca (*n *= 31)		
	*n*	%	*n*	%	*P* value
The medical unit or hospital is sufficiently equipped to attend high-risk pregnancies					
Agree	4	13.8	12	44.4	0.011*
Disagree	25	86.2	15	55.6	
The medical unit or hospital has the necessary medical personnel to attend high-risk pregnancies					
Agree	7	24.1	11	42.3	0.152
Disagree	22	75.9	15	57.7	
If required, vehicles (ambulances) are normally available or accessible in the medical unit or hospital for the transfer of patients-women with obstetric emergencies					
Agree	17	58.6	22	81.5	0.063
Disagree	12	41.4	5	18.5	
If required, the medical unit or hospital normally has or has access to communication services (radio, telephone, internet) that are necessary in the provision of medical care to patients with high-risk pregnancy or obstetric emergency					
Agree	14	48.3	20	74.1	0.048*
Disagree	15	51.7	7	25.9	
The medical unit or hospital provides me with sufficient training to care for high-risk patients					
Agree	12	42.9	14	53.9	0.419
Disagree	16	57.2	12	46.1	
The medical unit or hospital provides me with sufficient training to care for patients with obstetric emergencies					
Agree	8	28.6	13	56.5	0.044*
Disagree	20	71.4	10	43.5	
The medical unit or hospital provides me with sufficient training to care for patients from native communities					
Agree	6	21.4	11	44.0	0.079
Disagree	22	78.6	14	56.0	
There is an active policy of interaction in the treatment of the health of indigenous women between the health system and the beliefs of the native peoples					
Agree	7	28.0	11	47.8	0.156
Disagree	18	72.0	12	52.2	
The link between health services and the indigenous community is adequate					
Agree	14	53.9	11	45.8	0.571
Disagree	12	46.1	13	54.2	
The relationship between the clinic/hospital and the community facilitates the treatment of high-risk pregnancies of indigenous mothers					
Agree	24	85.7	16	69.6	0.163
Disagree	4	14.3	7	30.4	
In case of being required to attend an emergency or high-risk pregnancy of an indigenous woman, this clinic/hospital has the support of a translator					
Agree	2	7.4	9	39.1	0.007*
Disagree	25	92.6	14	60.9	

*P value of chi2 independence test statistic.

*Secondary level care (hospital care)*: At the time of labour and delivery, women must get to the hospital by their own means (from rural to urban areas, between 30 and 70 km); obstetric emergencies are transported by ambulance, if available at the health centre. In the qualitative interviews, most women agreed that accessing the hospital was problematic due to distance, the lack of accurate information to identify the specific point in pregnancy at which they should go to the hospital, and the lack of financial resources to pay for transportation. Upon arrival at the hospital, they encountered these inconveniences: errors of health care personnel in their assessment of the time to delivery; cases that were complicated by long transfer times and ended as emergency caesarean sections (34.1%; Table 4); no availability of ultrasound and other technological tools to aid diagnosis; missing medication and other medical supplies to adequately manage childbirth; and transfer to another hospital by their own means due to refusal of medical care when they arrived and requested service.

In contrast, the health personnel who were interviewed provided their version of the barriers and difficulties they have as professionals to attend to indigenous women as: language (they seek support from relatives for translation); adolescent pregnancy (10.7% of pregnant women in Oaxaca were between 15 and 19 years old and had complications, such as pre-eclampsia, twin pregnancies, and malnutrition, see Table 2); traditional beliefs and values (for example, authorities of indigenous communities prohibit family planning); saturation of hospital services; women’s late arrival for antenatal care, labour and delivery (most of the C-sections are due to prolonged labour, and only 9.4% were scheduled, see Table 1). One of the doctors interviewed reported this difficulty as follows:

“A large part of the population comes from neighboring villages, and they are communities with a (high) rate of marginalization, migration, (and) poverty. It is one of the poorest regions of the state and of the country. It is a population center where all that community is concentrated; all that population that comes from indigenous communities and arrives here and, even though they have hospitals … they come late for antenatal control … ” *(Doctor, Oaxaca)*

Other barriers that were identified include: topography of the region, with mountain villages six hours away from the hospital by road; transfer costs (women do not have money to pay for a taxi when the time for delivery arrives); political violence that restricts road traffic; and union strikes preventing the full operation of the hospital. In this model, few women receive care at childbirth with midwives; despite midwives’ training to identify obstetric emergencies, they are unable to address them and have to send cases to the medical unit. However, according to women’s survey responses, this model follows the regulations regarding post-obstetric event counselling; women said they received more information about: symptoms of bleeding, signs of pre-eclampsia/eclampsia, signs and symptoms of infections, thrombosis, nutrition, and handwashing. Few women reported going to private medical services (Table 4). On the other hand, the findings show that the federal inter-institutional agreement to care for obstetric emergencies which requires the participation of several institutions (social security, public and private), was not followed, as care was concentrated in only in two public health institutions.

*Specialised medical care*: Women who suffer complications in childbirth that cannot be treated in secondary care hospitals, are referred to the city of Oaxaca (172 km).

#### Model with international NGO participation

*Primary level care* is developed overall in the same way as the previous model. One difference is the incorporation of the respectful childbirth model, which includes antenatal care of pregnant women in the community by “companions” (women, volunteers from the community) who provide counselling on various issues related to sexual and reproductive health, and by the maternal health committee (which responds to obstetric emergencies). The NGO provides support for transfer with fuel vouchers or with their own vehicles. Their voluntary community health workers (known as companions) collaborated in five health centres and one “maternal health clinic” [*casa materna*], visited during the study. The NGO also provides infrastructure support (ensuring that ambulances are ready for transfer), medication, and personnel whom they train and supervise in technical aspects, interculturality matters, and quality of care. 97.9% of women surveyed stated that they began antenatal care during the first trimester of pregnancy, and 29% had deliveries managed at home or at the *casa materna*, accompanied by the midwife, health companions and obstetric nurses. Since the model has sought to incorporate new professional profiles in childbirth management and treatment, medical and nursing students – from public and private, local and foreign universities, who also participate in medical care – have been included. Women can choose who, including traditional midwives, treats them during childbirth; position for labour; non-pharmacological measures to control pain; breastfeeding immediately after childbirth, and family accompaniment throughout. This model focuses its efforts on the first level of care to concentrate the demand on obstetric services and reduce hospital saturation. Only cases with medical complications are referred to the hospital. (Figure S2 in Supplementary material describes the structure of this model.)

*Secondary level care (hospital care)*: Primary health care centres can refer obstetric emergencies to the general hospital in the nearest city (two hours away, by road). On average, 89.7% of the women had normal, physiological childbirth mainly at the *casa materna*. However, some (36.6%) had complications during pregnancy, and seven women had emergency caesarean sections from these. Other notable results show that in Chiapas women had more accompaniment during the birth process (33.0%), while 24% required post-obstetric event counselling. Regarding the remaining indicators under this category, a smaller number of the women surveyed in Chiapas received information compared to the women in Oaxaca (Table 2).

*Specialised medical care.* A general hospital with the capacity to respond to and treat emergencies requiring medical specialties was three hours away by road. None of the respondents reported having used it.

### Quality of care indicators in both models

#### Reported by health care personnel

*Infrastructure*: There was agreement on the lack of equipment (Chiapas 86.2%, Oaxaca 55.5%), and personnel to attend to high-risk pregnancies and obstetric emergencies (Chiapas 75.9%, Oaxaca 57.7%) (Table 5).

*Training*: More than 50% of the respondents in Oaxaca agreed that they are trained to attend high-risk patients and patients with obstetric emergencies, and that they have adequate communications services (telephone, internet). In contrast, the respondents in Chiapas did not agree with this (Chiapas 71.4%, Oaxaca 43.5%, statistically significant differences *p *= 0.044). Regarding specific aspects of attending to and treating the indigenous population, 78.6% said they felt less prepared, compared to Oaxaca (56%); similarly, they did not consider that there were active health policies for understanding the local culture: Chiapas 72%, Oaxaca 52.2% (Table 4).

*Privacy*: In the Chiapas model, women can be accompanied by a family member or relative during childbirth. However, not all the health personnel like the idea; some considered that it violates the privacy of other users, because the spaces are not large enough. In comparison, that possibility is not available in Oaxaca; women are admitted to the hospital, and the family and relatives must wait for them outside. A doctor from the Chiapas model commented on this matter as follows:

“ … If we have three, or two women in labour in the room, and there can be no privacy between one and the other, it is better not to have a companion because he or she will see the other woman in there. It depends on the situation; usually, if there are two or three, we do not let companions in the room, so the women in labour don’t feel bad.” *(Doctor, Chiapas)*

*Freedom of choice*: In the Chiapas model, the role of the health care personnel is to provide information and alternatives for the users. Freedom of choice is evidenced during labour in the *casa materna*, where women can choose the labour position and the place for delivery. However, there were also disagreements among the health personnel regarding giving women the opportunity to make those decisions. For example, respondents of the Chiapas model felt that the choice of labour position would be much easier if women received this information during the antenatal stage, since once labour begins it is difficult for women to make decisions due to pain and stress. Another example is the choice to be assisted by a midwife, which they did not oppose, but felt that a woman opting for a midwife should receive prior information about the risks that may be involved, such as lack of transportation to take her to the *casa materna* if complications occur during childbirth. In comparison, these options are not available in the Oaxaca model; women are treated in a single way and they are not able to choose labour position or place, or opt for the type of professional they want for childbirth.

“We had a case in which the pregnant woman wanted a midwife to be with her during labour and childbirth; they talked to the doctor, and to the hospital director, and she did give authorisation … ” *(Nurse, Chiapas)*

*Timely care*: In Oaxaca, the main difficulty pointed out by health personnel was the lack of ambulances to transport women. Additionally, they stated that women need to be trained to identify risk factors and know when to see a doctor. In comparison, in the Chiapas model, when women have some complication of childbirth, the “companions” support them in the search for medical care.

“People were afraid to go to the hospital because they were not treated right away; they would have the baby [give birth] in the hallway, then no one would come and check them, they would just leave them there, so what people would do is give birth with a midwife instead. Thank God that now our fellow health providers are doing their job well at the hospital … . Almost all the women are now going to the hospital, we give them their birth plan and fill out a form … and they go down to the hospital.” *(Companion, Chiapas)*

#### Reported by women

*Infrastructure*: Experiences of childbirth care were diverse, depending on the condition of each pregnant woman. In the Chiapas model, primiparous women with low-risk pregnancies, who did not have previous experiences, perceived delays in care due to lack of human or material resources. There were even those who preferred to look for the midwife on their way back home. One of the women commented:

“At the beginning they received me and treated me right away … they took me to the waiting room, but at the time of delivery they didn’t, because they were not minding their job, they [the health care personnel] were doing something else. I do not think that my little girl caused the tear because she was very little, she weighed 2.300 kg and when they stitched the tear, it hurt more; they did not use anesthesia, and they [couldn’t do it]; they [gave] up … and they sent me to Villaflores.” *(User, 23 years old, perineal tear, Chiapas)*

However, in the Chiapas model, when it came to women with children, the perception regarding the care provided was different; they believed that the medical units did have enough supplies to provide proper care. In comparison, in the Oaxaca model, most of the stories reported insufficiency of: beds to receive them and treat them; health care personnel; medication; ultrasounds; and reagents for laboratory tests, among other medical supply shortages. Additionally, the women explained that they did not receive adequate gowns, towels and bed sheets to cover themselves and their newborn babies, which also affected the issue of privacy during medical observation.

“Well, I felt scared and sad at the same time because I got scared, because I thought ‘if they do not treat me I am going to have this baby outside' [in the street] and since someone had already told me that just recently another lady had had her baby outside … I kept thinking the same would happen to me … I even slept outside for a little while, because they didn’t tell me: ‘well, stay there if you already have labour pains'.” *(User, Oaxaca)*

*Privacy*: In both models, the women agreed that they did not have enough privacy during the observation of the active phase of labour, since inside the rooms, the space was divided only by curtains.

“There were other two ladies besides me, the three of us were in the hall, and we could see each other, we would pull down the curtain and we could see each other; I would suggest that they have more rooms so that we could all go there; more rooms, more privacy. […] I am a first timer, and I feel nervous and embarrassed; other ladies already have 3 or 4 children and they would just open the curtain and I would say: No! because I was just in my gown and such.” *(User, 21 years old, Chiapas)*

*Freedom of choice*: The Chiapas model is based on freedom for users to choose who will attend the delivery. This was referred to as something positive by most of the interviewees: they could receive childbirth care in their village with the midwife, in the maternal health clinic with a midwife or medical staff. The choice of the position for labour and childbirth was part of the promotion of freedom of choice for women; the majority reported having been able to adopt the position of their choice. They were also able to choose family planning methods in an informed manner, and they recognised the ease of obtaining these in health centres or in the c*asa materna*.

“They took me to the bed where they had already checked me and then they got me out of there and told me how I was going to have my baby and [I asked about] possibilities, and they told me, and then they asked me how I wanted to have my baby, and I said: ‘I want to be kneeling down'.” *(User, 30 years old, Chiapas)*

In comparison, in the Oaxaca model, once women are admitted to the hospital for childbirth care they lose all freedom of choice. Childbirth care was always performed under the medicalised model, and the health personnel’s insistence on a method of permanent birth control was one of the main complaints against this model of care.

*Timely care*: In relation to transfer and waiting times, the average transfer time for Oaxaca was 1.3, and 1.8 h for Chiapas; however, waiting times on arrival at the hospital were 2.1 h for Oaxaca and 0.5 h for Chiapas (see Chart 1 in S3 Supplementary material). In the Oaxaca model, despite the existing agreements between hospitals to deal with obstetric emergencies, several women reported that they were not admitted for childbirth, even in an emergency.

*Treatment*: The women surveyed reported that they felt mistreated by health workers in both models of care, although the percentage was higher in Oaxaca (27.8%) than in Chiapas (14.8%). It is notable that, in Chiapas, 91.3% of the women were informed about their medical condition and the procedure to be followed, in contrast to Oaxaca, where only 78% were informed. In Chiapas, all the women who received information said they understood it; in Oaxaca, only 76.7% said so, and 11% said that they needed a translator. However, there was a higher percentage of informed consent before childbirth in Oaxaca than in Chiapas (85.7% vs 71.3%), and also with regard to the delivery of a medical report upon discharge (91% vs 76.5%). Loss of files occurred in 3.2% of the women surveyed in both sites, and only 71% knew that they could complain if they did not agree with the treatment. Thus, approximately one in three women were not aware of the fact that they could complain and, even when they know, the submission of complaints is not a frequent practice (Table 6).

**Table 6.** Characteristics of the quality of care by model of care^a^

	Chiapas		Oaxaca		Total	
	*n*	%	*n*	%	*n*	%
Felt mistreated by health personnel *	17	14.8	37	27.8	54	21.8
Was informed about her condition and the procedure to follow *	105	91.3	104	78.2	209	84.3
Understood everything the medical staff said to her *	105	91.3	102	76.7	207	83.5
Needed a translator to understand the explanations of the care process^b^	0	0	7	11.3	7	11.1
Has lost her file or documents at some point	3	2.6	5	3.8	8	3.2
Was asked for her consent before the birth procedure *	82	71.3	114	85.7	196	79
Was given a medical report about her health status *	88	76.5	121	91.0	209	84.3
Received the birth certificate of her child	109	94.8	131	98.5	240	96.8
Knows that she can complain if she does not like the way she is treated	86	74.8	90	67.7	176	71

^a^This table does not include information on deliveries performed at the woman’s or the midwife’s home.

^b^It only includes women who speak an indigenous language.

*P value of chi2 independence test statistic.

*Shelter*: Both models of care have a place where both women and their families can wait for the time of childbirth, which seeks to reduce risks in transfer from their villages and facilitate monitoring. According to data from the survey, in Chiapas, 42% of the women reported having spent the night in the shelter or having slept in a place other than their usual residence before childbirth. Most of the women reported having received timely childbirth care and the offer of accommodation at the c*asa materna* for themselves and a companion. A user from Chiapas described it as follows:

“I was treated that same afternoon, they [asked me] where I lived, and they said to me: ‘there is not enough time for you to go back … don’t leave', they told me. They performed a pelvic examination, then they checked how my baby was. If your baby is not born tonight [then], he’ll be born tomorrow, they said.” *(User, 14 years old, Chiapas)*

In comparison, in Oaxaca, although the shelter was made to support those who came from more remote communities, it was not easy for them to use it as it was always occupied.

## Discussion

Mexico failed to reach the millennium development goals in relation to the reduction of maternal deaths for 2015 and continues to lag significantly.^28,29^ It is well known that disadvantaged, rural and indigenous populations contribute the greatest number of these deaths annually, and they are also the populations with the greatest shortfall in provision of care for pregnancy, childbirth and the puerperium.^3,30^ The results of the study presented in this document show concrete and specific elements that can help explain the outcomes of studies based on national statistics.^19^ We seek to show the problematic aspects related to the quality of care in two similar population contexts, yet with different models of care.

We selected two areas of study that, in theory, have created health service networks using available physical, material and human resources to improve access for vulnerable populations. One area applies the model of medicalised treatment that is commonly used in most public health services in Mexico. In the other, a civil society organisation collaborates with the medicalised model while practising “respectful childbirth”,^31^ seeking to empower women to make choices throughout the pregnancy-childbirth-puerperium process and promoting alternative practices to the traditional model, based on scientific evidence.^32^

The study compares the opinions of service providers and users on a set of indicators of quality of care. These opinions are shared, in general, with regard to the low availability of technological, pharmaceutical and human resources in the units, and the difficulties women face to travel from their villages to hospitals and to find the necessary resources to pay for such expenses. On the other hand, there are important contrasts in relation to the behaviours and attitudes of service providers towards the users, and the way in which they are trained and in which they apply the official standards of mandatory compliance. However, it is worth noting that, in both models, the health service providers complained about not having adequate conditions in terms of infrastructure, nor being sufficiently trained and equipped to properly serve indigenous women; this issue reveals another focus for the health care system, in the sense that technical personnel cannot be expected to solve situations for which they have not been trained, such as the cultural reasons women have for not seeking care in the hospital or for seeking it with other types of health providers. These types of contrasts have already been reported in previous studies.^33,34^ Notwithstanding, the obstetric violence reported by women transgresses the concept of quality of care*;* it is an issue that has already been reported in Mexico and in other countries,^35^ although in the case of indigenous women, it is a phenomenon that is exacerbated.^36^ These women continue to experience racism and discrimination in different aspects of social life related to public institutions.^37^

Institutionalised obstetric violence has a wide range of manifestations, some implicit and others explicit. It is worth noting that health providers do not recognise this as a general problem, except for specific cases. However, the women’s survey responses and the elaboration of their opinions in the qualitative study show different angles of this phenomenon. Three aspects can be highlighted in both models: freedom to choose, the waiting times before receiving treatment, and the treatment by staff.

Women’s freedom to choose is a key component of respectful childbirth.^32^ In the Mexican public health care system, this concept is completely new for health personnel, particularly doctors, who continue to use procedures that are not recommended (or whose application is restricted) by the WHO, such as episiotomy, umbilical cord traction, or caesarean section, and thereby violate women’s rights. The women did not have specific preferences regarding these practices as they do not know that they are not recommended; however, they do have preferences regarding the position in which to give birth when they are given the option, an option that is not allowed in the medicalised approach to childbirth care, which lacks the innovative vision of the alternatives incorporated in Chiapas. Respectful childbirth also implies that the provision of care should be focused on the women and not on the service providers or on the institution itself. We observed that, in Oaxaca where childbirth is managed under the medicalised model, waiting times for the provision of care once a woman has arrived at the medical unit are four times higher than in Chiapas (see Chart 1 in S3 Supplementary material), where the respectful childbirth approach has been introduced. This could imply that the reduction in waiting time is more related to the attitude of the health care providers, putting women at the centre of the care to be provided, than to the availability of resources, theoretically increased by the existence of an inter-institutional network of services.^38^

Finally, manifestations of abuse were reported by one fourth of the women in Oaxaca, and one eighth of the women in Chiapas. Although verbal and physical abuse during the use of obstetric services has already been reported in México,^39^ it is clear that turning the focus of medical care onto women, as in Chiapas, reduces it. Another related element is that more women in Oaxaca than in Chiapas stated that they only speak an indigenous language. This constitutes a profound obstacle in the interaction with health care personnel that is not fully resolved with translation, since it poses the need for greater cultural integration between the health system and health care providers with the indigenous populations of the country.

It is worth noting that during the data collection period in Chiapas, the implementation of the respectful childbirth model had just begun in the maternal health clinic, so the results obtained from there still reflect a transitional phase. Implementation at the maternal health clinic has now been completed; in 2018, all deliveries were managed under this model by obstetric nursing students, with supervision. The implementation of the model has also made progress in the referral hospital, but with difficulties due to the resistance of doctors to learn and implement it. However, as indicated above, the implementation of the model in Chiapas offers important benefits for indigenous users. In Oaxaca, we have no evidence that the implementation of the respectful childbirth approach has begun in the communities studied.

Government changes to public health policies in Mexico are in a new stage, proposing to strengthen the primary level of care, encouraging a community approach in health centres, and to generate a pluralistic offer of health service providers within the medical units. Nevertheless, as this article demonstrates, indigenous women require special attention to their sexual and reproductive needs, both objective and subjective, including different ways of understanding health, sickness, reproduction, and parenting. Of fundamental importance is respect for their reproductive preferences through a user-centred model of care; experience in other countries has shown how this benefits women.^40–44^

## Conclusions

Ensuring universal access to health care services for the poorest, most vulnerable population is one of the sustainable development objectives of the United Nations 2030 agenda; in other low- and medium-income countries, this has been accomplished by harmonising national agendas with international commitments.^45,46^ Universal health coverage advocates access for the most disadvantaged, vulnerable populations as a priority.^46–48^ However, the issue of *access* has problems related to the effect of certain structural social determinants, which limit: arriving at the site of service, following medical recommendations, being referred to the next level of medical care, long waiting times for care, understanding medical recommendations, paying for laboratory tests, paying for the medication required for treatment, tolerating abuse experienced during childbirth, refused admission, and pilgrimages from one hospital to another.^19^ In Mexico and other countries around the world, universal access to health care is currently being considered;^49,50^ however, our findings show that this goal requires actions that go beyond the development of regulations, as it is evident that health personnel may not follow current norms and standards and may ignore institutional agreements.^34^ Therefore, it is necessary to modify the structure of organisational values in order to place the user at the centre of medical care and ensure respect for their rights. The participation of agents outside the public system (such as NGOs) can be of great value to move in this direction. It also requires the participation of women, to learn about the way in which they are being cared for, and the extent to which they are satisfied with the obstetric services. This research experience can be used for other countries with similar circumstances.

## Limitations of the study

Because this is an observational study, the above is exclusive to the reality studied and is not intended to formulate generalisations. The cases that were selected are not strictly comparable, so the inferences that are hereby raised have a value that is limited to the cases themselves and not to other situations in the country.

## Supplementary Material

S1, Figure 1

S2, Figure 2

S3, Chart 1

## Footnotes

* In an article published previously, we analysed some of the responses of women from Oaxaca regarding their perceptions of access barriers to health services, using the model of T Tanahashi, which suggests studying access through these dimensions: availability, accessibility, acceptability, and continuity of service. For quantitative analysis (*n *= 149), we analysed sociodemographic variables (age, marital status, occupation) and other variables related to the maternal and perinatal health care process, i.e. doctor-patient relationship (treatment received), and organisation and infrastructure of the health care services and facilities (waiting times, free medication, delivery care, complications). For the qualitative analysis, 30 cases were selected, and the aforementioned dimensions of the T. Tanahashi model were inferred from the issue of care quality.^21,26^
