# Supplementary material for: Lags in the provision of obstetric services to indigenous women and their implications for universal access to health care in Mexico
Source: Sex Reprod Health Matters. 2020 Aug 6;28(2):1778153. doi: 10.1080/26410397.2020.1778153 (PMC7888012; doi:10.1080/26410397.2020.1778153)
Supplement: S1, Figure 1 [file ZRHM_A_1778153_SM0218.docx]

**SUPPLEMENTARY MATERIAL**

**S1. Figure 1. Structure of the Oaxaca healthcare model.**

Health Assistants

Auxiliares de Salud

Health Clinic

(Community Brigade)

Health Clinic

(Community Brigade)

Health Clinic

(Brigade)

Health Clinic

(Brigade)

Health Clinic

(Brigade)
