# Supplementary material for: Lags in the provision of obstetric services to indigenous women and their implications for universal access to health care in Mexico
Source: Sex Reprod Health Matters. 2020 Aug 6;28(2):1778153. doi: 10.1080/26410397.2020.1778153 (PMC7888012; doi:10.1080/26410397.2020.1778153)
Supplement: S2, Figure 2 [file ZRHM_A_1778153_SM0214.pptx]

## Slide 1
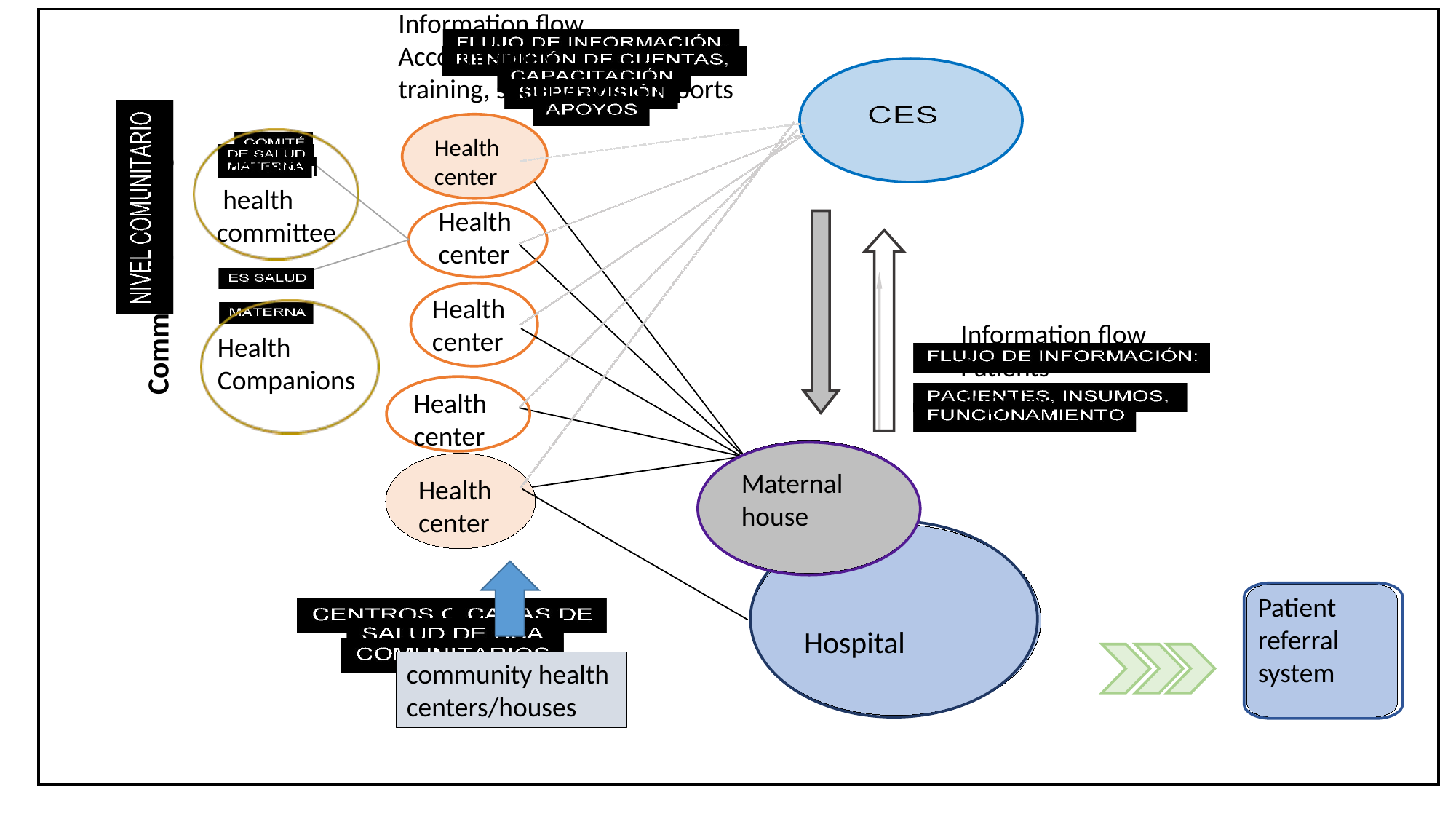

Information flow
Accountability
training, supervision, supports
Health
center
Health
center
Maternal
house
Health center
Hospital
Patient referral system
Maternal
 health
committee
Community setting
Health
center
Information flow
Patients
Supplies
Health
Companions
Health
center
community health
centers/houses
