# Supplementary material for: Lags in the provision of obstetric services to indigenous women and their implications for universal access to health care in Mexico
Source: Sex Reprod Health Matters. 2020 Aug 6;28(2):1778153. doi: 10.1080/26410397.2020.1778153 (PMC7888012; doi:10.1080/26410397.2020.1778153)
Supplement: S3, Chart 1 [file ZRHM_A_1778153_SM0198.docx]

**SUPPLEMENTARY MATERIAL**

**Chart 1. Transfer and waiting times**.
